# Supplementary material for: A Highly Expressing, Soluble, and Stable Plant-Made IgG Fusion Vaccine Strategy Enhances Antigen Immunogenicity in Mice Without Adjuvant
Source: Front Immunol. 2020 Dec 4;11:576012. doi: 10.3389/fimmu.2020.576012 (PMC7746858; doi:10.3389/fimmu.2020.576012)
Supplement: Supplementary file 1 [file DataSheet_1.pdf]

Supplemental Figures

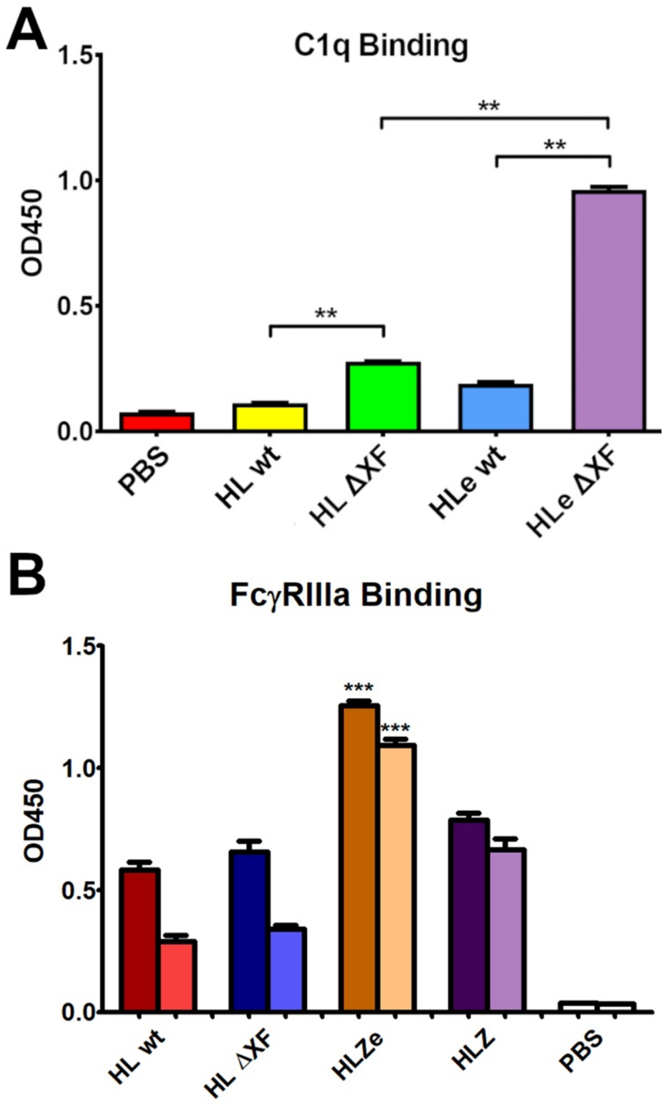

Figure S1. C1q and FcγRIIIA binding of constructs.
(a) ELISA plates were coated with 10 µg/ml human C1q and incubated with 5 µg/ml each purified construct. Constructs were detected using polyclonal goat anti-human IgG-HRP. Mean OD_{450} values from three replicates are shown ± standard error with two stars indicating $p < 0.01$ as measured by one-way ANOVA with comparisons between the indicated groups.
(b) ELISA plates were coated with 1.2 µg/mL of human FcγRIIIA and incubated with purified constructs at equimolar concentrations; right bar for each construct is 10-fold dilution of the left bar. Constructs were detected using goat anti-human IgG (whole molecule) -HRP. Mean OD450 values from three replicates are shown ± standard error; three stars (***) indicate $p < 0.001$ as measured by one-way ANOVA comparing each HLZe dilution to the respective dilutions of each other construct. Abbreviations: wt, constructs made in wildtype *N. benthamiana* plants; ΔXF, constructs made in glycoengineered plants silenced for xylosyltransferase and fucosyltransferase.

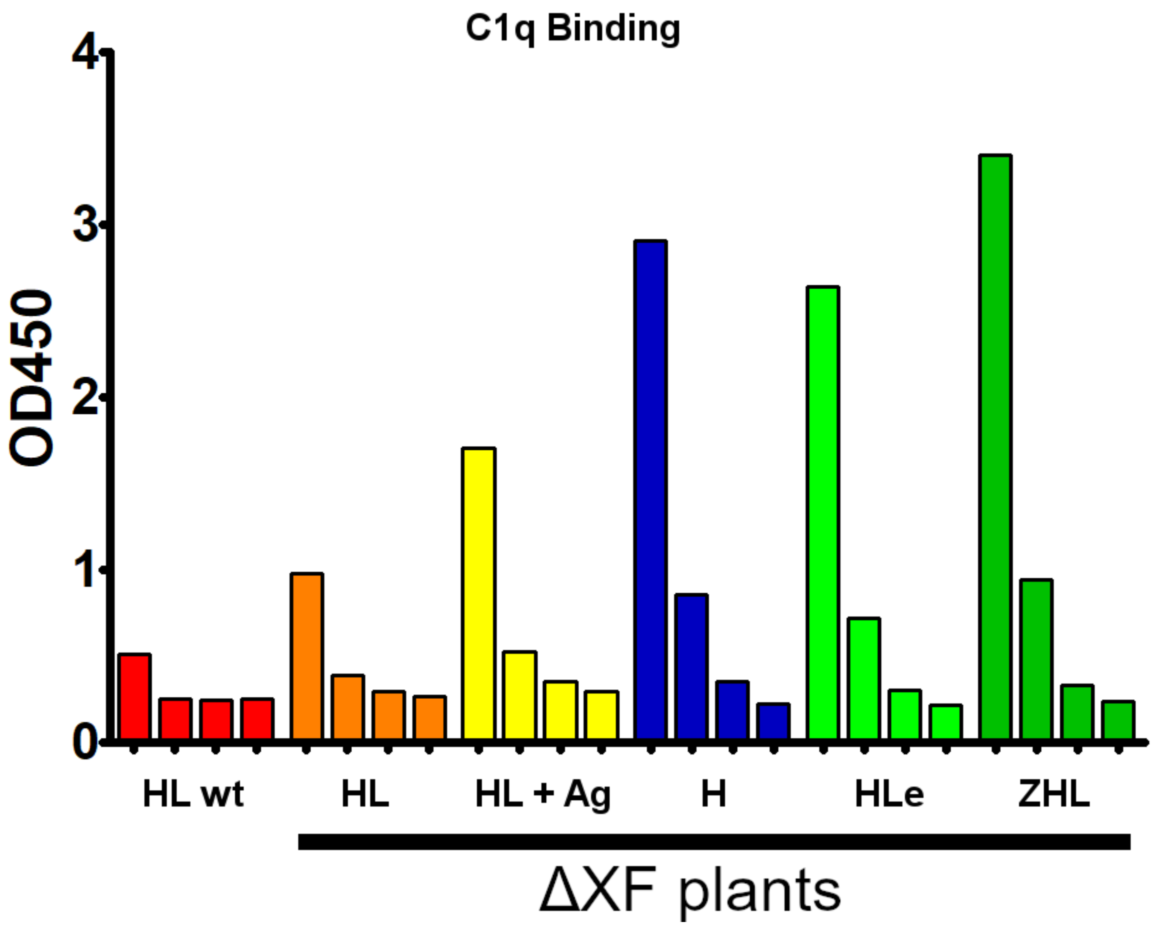

Figure S2. C1q binding comparison between IgG fusions
ELISA plates were coated with 10 μg/ml human C1q and incubated with 10-fold serial dilutions of each purified construct starting at 5 μg/ml. Constructs were detected using polyclonal goat anti-human IgG-HRP. Mean OD_{450} values from three replicates are shown. Abbreviations: wt, constructs made in wildtype *N. benthamiana* plants; ΔXF, constructs made in glycoengineered plants with reduced xylosyltransferase and fucosyltransferase activities; Ag, dengue cE tagged with the 6D8 epitope; ZHL, same as HL but with ZE3 fused to the N-terminus of the 6D8 heavy chain.

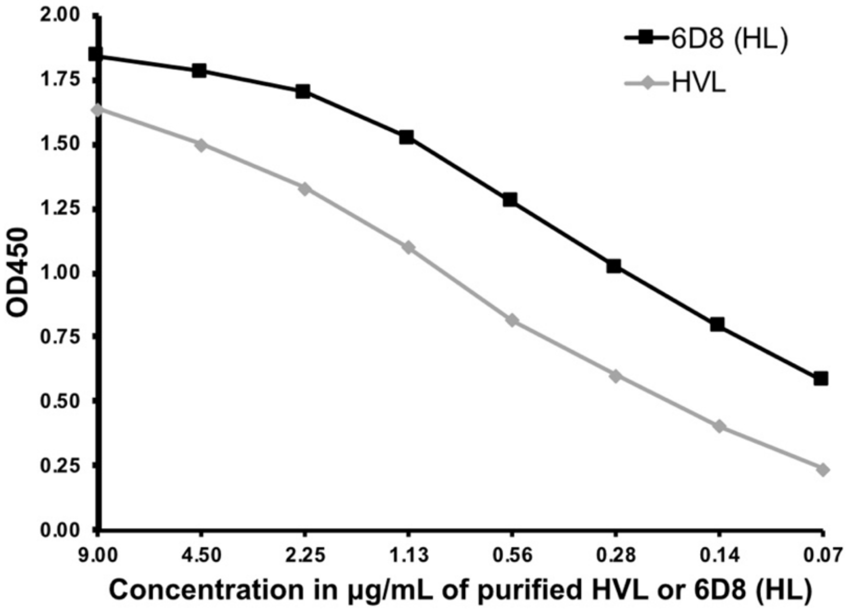

Figure S3. Epitope binding of single chain 6D8
ELISA plates coated with 900 ng of purified epitope-tagged protein were incubated with serial dilutions of either HVL or full-length 6D8 antibody. The bound constructs were detected with HRP-labeled mouse anti-human IgG (Fc-only) antibody and the absorbance was read at OD_{450}. Abbreviations: HVL, single-chain antibody with the variable heavy chain linked to the variable light chain region that is fused to the 6D8 heavy chain constant region; 6D8, full-length antibody containing both heavy and light chains.

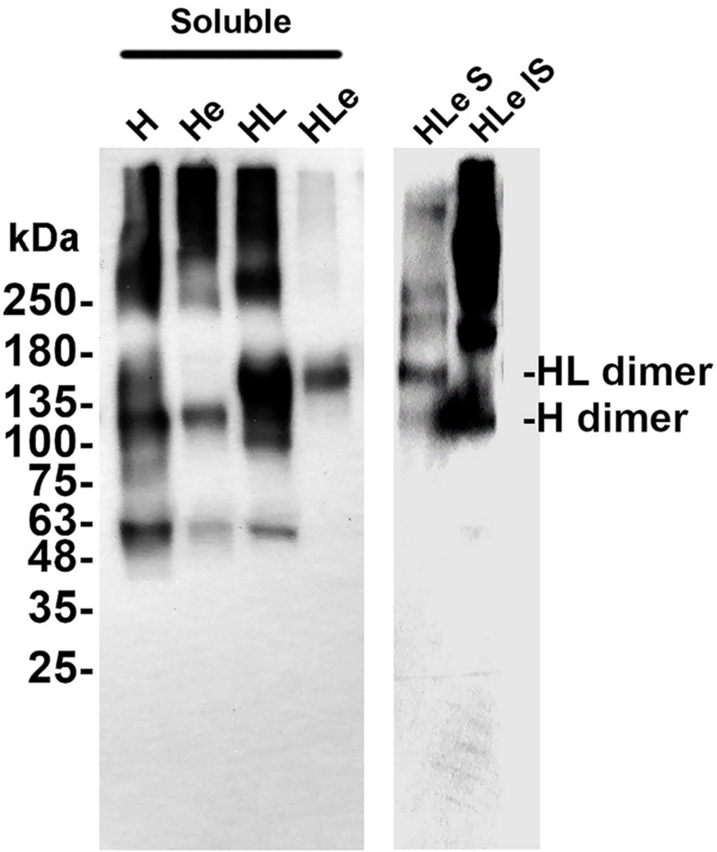

Figure S4. RIC insolubility

Protein was extracted from leaves of *N. benthamiana* agroinfiltrated with the indicated constructs and separated by SDS-PAGE followed by western blotting under nonreducing conditions. The constructs were detected using goat anti-human IgG-HRP. Soluble (S) refers to the clarified crude leaf extract, while insoluble (IS) refers to the pellet following clarification that had been resuspended in SDS sample buffer.

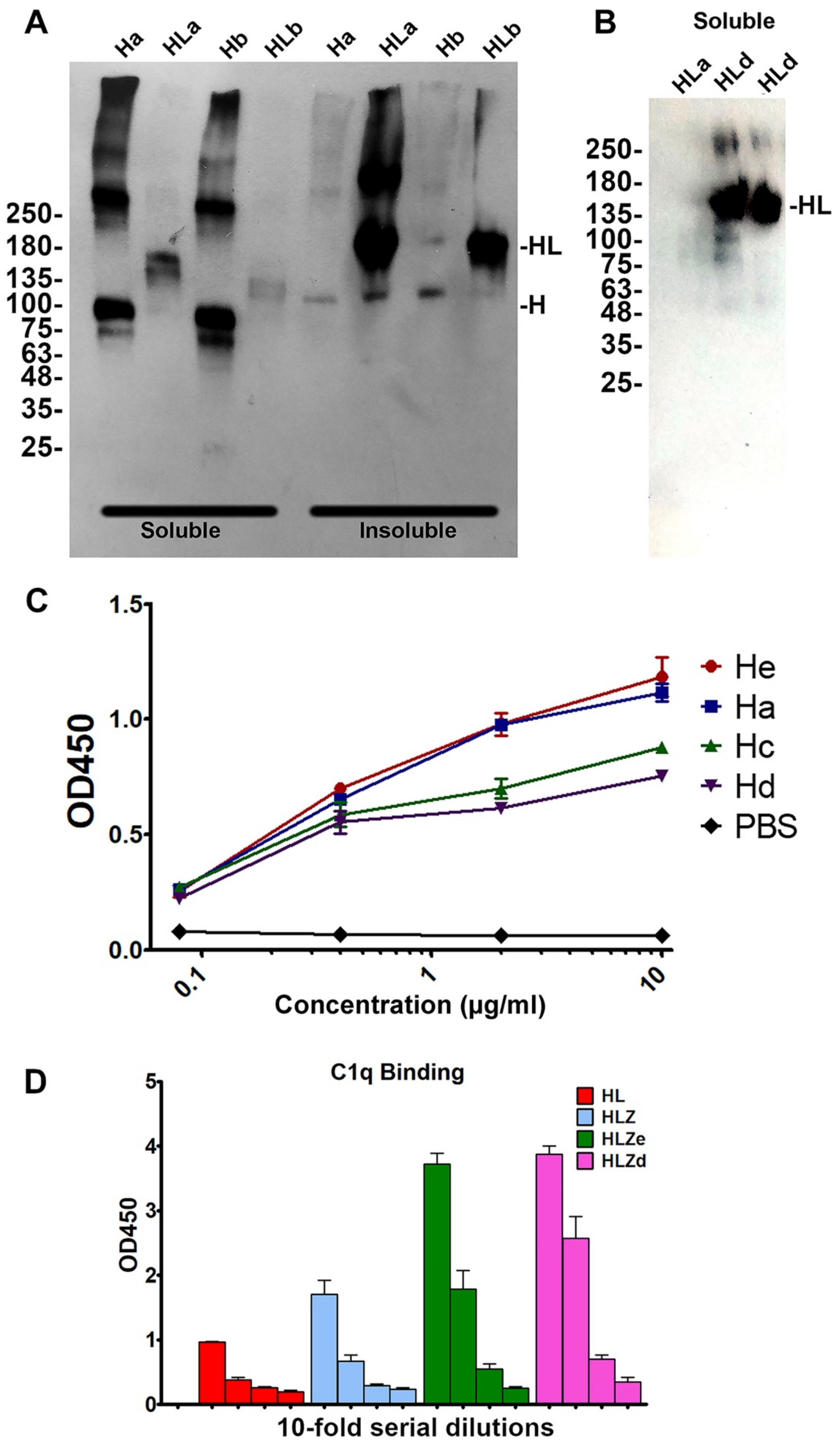

A
Ha
HLa
Hb
HLb
Ha
HLa
Hb
HLb
250-
180-
135-
100-
75-
63-
48-
35-
25-
-HL
-H
Soluble
Insoluble
B
Soluble
HLa
HLd
HLd
250-
180-
135-
100-
75-
63-
48-
35-
25-
-HL
C
OD450
1.5
1.0
0.5
0.0
0.1
1
10
Concentration (µg/ml)
He
Ha
Hc
Hd
PBS
D
C1q Binding
HL
HLZ
HLZe
HLZd
OD450
5
4
3
2
1
0
10-fold serial dilutions

Figure S5. Solubility and binding of 6D8 epitope tag mutants

(A, B) Protein was extracted from leaves of *N. benthamiana* agroinfiltrated with the indicated constructs and separated under nonreducing conditions by SDS-PAGE followed by western blotting using goat anti-human IgG-HRP as probe. Soluble refers to the clarified crude leaf extract, while insoluble refers to the pellet following clarification that had been resuspended in SDS sample buffer. The epitope mutant designated "a" contains epitope sequence VYKLDISEA; the epitope mutant designated "b" contains epitope sequence VYKLDISE; the epitope mutant designated "c" contains epitope sequence YKLDISE; the epitope mutant designated "d" contains epitope sequence YKLDIS. (C) ELISA plates coated with serial dilutions of purified heavy chains containing the indicated epitope tag mutants were probed with full-size 6D8 followed by goat anti-human kappa-HRP. Mean OD_{450} values are shown from three replicates ± standard error. (D) C1q binding of 10-fold serial dilutions of epitope mutant HLZd compared to full epitope tag HLZe. Note the 10-fold increase of both constructs compared to HLZ (no tag).
